# Multiple sclerosis: As we see it today and glimpses of tomorrow

**DOI:** 10.4103/0972-2327.58271

**Published:** 2009

**Authors:** Kottil W. Rammohan

**Affiliations:** Professor of Neurology, The Ohio State University, Ohio, Columbus, Ohio, USA. E-mail: rammohan.2@osu.edu

In the Western hemisphere, multiple sclerosis is a common neurological disorder. It is less common in the East, and in addition, the disorder also has a predilection to temperate over equatorial regions. There is emerging evidence that this disorder may be changing, affecting regions and populations that were previously considered less vulnerable. Is it just that we have become better at diagnosing this disorder or is there a real change in prevalence? There is much evidence that this is not simply a matter of better disease ascertainment, but that instead the world-wide incidence, prevalence, gender differences, and severity of this disorder are all changing. India is no exception. From what used to be a relatively rare disorder, this disease is emerging as a disorder that is no longer uncommon. Although the prevalence of multiple sclerosis in India is still less than that in the Americas and Europe, multiple sclerosis is a disorder that affects virtually every ethnic group within India.

The goal of this supplement is to bring to the reader greater awareness of the many advances made in this disorder, particularly in the last two decades. Advancements in immunology and genetics have furthered our understanding of the pathogenesis of this disorder, and the state of the art concepts in pathogenesis and genetics can be discerned from the chapters on these topics by Drs Racke and Sawcer, respectively.

Is the problem entirely genetic or is there an environmental trigger? The chapter by Dr. Sriram on the infectious basis of multiple sclerosis examines current concepts concerning the role of an environmental trigger.

In recent years, it has become evident that neuromyelitis optica is more than optic neuritis and transverse myelitis and is established as a unique inflammatory disorder of the nervous system distinct from multiple sclerosis. I owe a special thanks and gratitude to Dr Anu Jacob who was recruited late to write the chapter on this disorder, but nevertheless produced on time a masterly discussion on this complex and evolving disorder.

The chapter by Dr Barrie Hurwitz elucidates the current diagnostic criteria on multiple sclerosis, followed by my chapter on the utility of cerebrospinal fluid and Dr Henry McFarland's discussion of the use of magnetic resonance imaging in multiple sclerosis.

Treatment of this disorder is covered by a number of contributors. Dr Alexander Rae-Grant has out-lined the treatment of patients with acute exacerbations. Prevention of exacerbations by current disease modifying agents is covered by Dr Patricia Coyle. The emerging role of anti-B cell therapies is elucidated in the paper by Dr Kathleen Hawker. Potential future agents for treatment of this disorder are described by Dr Calabrese and his colleagues. Maintaining quality of life in the patient already afflicted by MS is the corner-stone of treatment of this disorder, and Dr Randall Schapiro and Dr Jack Burkes have expanded on symptom management and the role of rehabilitation in our patients, respectively.

It is especially disheartening when children are affected with multiple sclerosis and in recent years pediatric multiple sclerosis has become recognized as an entity in itself. Children are not just young adults, and the disorder has issues and ramifications that go beyond the problems seen in adults. The chapter by Dr Lauren Krupp examines these problems in this population.

Great insights into the pathogenesis of MS can be learned from children who represent a population relatively naive to pathogen exposure. In some respects, the population of patients with multiple sclerosis in India is like the pediatric population, namely an emerging disorder in an otherwise multiple sclerosis naive population. The study of this disorder in India can offer insights into this disease in a manner not possible in areas where this disorder is common. It is my hope that this supplement will spawn such interests and trigger studies on this disorder where we are desperately seeking answers on the etiology, pathogenesis, and treatment.

The supplement is broad in its scope but by no means a complete compendium on multiple sclerosis. It has just skimmed the surface of the enormous volumes of available information. If the reader finds these articles sufficiently stimulating to desire more in-depth information on the topics, the purpose of this supplement will have been served.

Lastly, I owe each author a personal debt of gratitude for his or her contribution, without which this supplement would not be possible. This supplement is dedicated to you and our fellow physician scientists who endure the trials and joy that come with the care of our patients, and for our patients who must endure this disease with the trials but not the joy of suffering from this disorder.

